# Non-Surgical Periodontal Defect Fill and Spontaneous Tooth Repositioning: A Case Report

**DOI:** 10.3390/reports9020186

**Published:** 2026-06-15

**Authors:** Hani T. Fadel

**Affiliations:** Division of Periodontology, Department of Preventive Dental Sciences, College of Dentistry, Taibah University, Al Madinah Al Munawwarah 42353, Saudi Arabia; hani.fadel@yahoo.com; Tel.: +966-(0)-5-5440-6095

**Keywords:** alveolar bone loss, dental scaling, periodontitis, root planing, tooth migration

## Abstract

**Background and Clinical Significance**: Advanced periodontitis destroys tooth-supporting structures and causes pathologic tooth migration (PTM) and functional and esthetic repercussions. This report illustrates how non-surgical periodontal therapy (NSPT) addresses such concerns. **Case Presentation**: A 34-year-old woman (healthy, who quit smoking) complained of a space between her upper front teeth. Based on clinical and radiographic data, she had localized periodontitis stage III grade C with intrabony defects, deep pockets, and a diastema between teeth # 12 and 11. The treatment plan involved improvement of self-performed oral hygiene measures and NSPT. Initially, improvements in plaque, bleeding, and pocket depth scores were observed. Non-surgical re-treatment of residual sites was associated with further improvements and complete closure of the diastema, along with patient satisfaction with the outcome. **Conclusions**: The potential of NSPT in improving periodontal clinical parameters and the association with spontaneous tooth repositioning and apparent filling of intrabony defects radiographically is demonstrated, ultimately addressing the patient’s functional and esthetic concerns.

## 1. Introduction and Clinical Significance

The treatment of various oral conditions has evolved rapidly over the years. From the application of complex surgical procedures, through the use of advanced materials science, to the utilization of innovative technological breakthroughs [1,2,3,4,5,6]. Historically, every single evolutionary step supposedly aimed to enhance treatment outcome, reduce procedural time, decrease required costs and/or improve the patient’s experience as a whole.

Periodontitis constitutes a multifactorial clinical manifestation, as it results from an inflammatory reaction to microbial plaque/biofilm and involves loss of vital tooth-supporting structures, which may eventually lead to tooth loss if left untreated [7,8]. This is associated with impacts on function, esthetics, the overall health and the quality of life [9,10]. The treatment of periodontitis is relatively straightforward and is cause-related in principle, as it involves consistent removal of the biofilm (and biofilm-bearing deposits, i.e., calculus) from the tooth surfaces by both the patient and the dental professional [11]. Non-surgical periodontal therapy (NSPT)—also known as scaling and root planing (SRP)—is the initial and standard approach employed by specially trained dental professionals or periodontists and involves the careful mechanical removal of calculus from the teeth roots [11]. The literature shows that NSPT is generally successful in improving clinical parameters such as bleeding on probing (BoP), periodontal probing pocket depth (PPD) and clinical attachment level (CAL), in addition to the patient-centered outcomes [11]. Chemical means are also used as adjuncts to aid in minimizing clinical symptoms [12]. However, tooth-supporting structures that were lost as a result of the destructive nature of periodontitis often require additional procedures to restore them in their entirety or at least in part. Regenerative periodontal therapy refers to surgical procedures that would lead to periodontal regeneration and often involve the use of certain material(s) or agents in the space or defect resulting from loss of the periodontal structures [13]. Ideally, these procedures would achieve true periodontal regeneration, which is a biological term inferring the histologic reconstitution of new alveolar bone, periodontal ligament and cementum over a root surface previously deprived of the attachment apparatus [14]. This will naturally be accompanied by improvement in clinical parameters and would appear positively on diagnostic radiographs. However, clinical and radiographic improvements alone cannot confirm the establishment of periodontal regeneration; thus, the outcome is appropriately referred to clinically as periodontal reconstruction [14]. Occasionally, “defect-fill” is observed on radiographs and suggests the restoration of the lost bone in the affected defect [13]. Despite clinical improvements, pre-clinical studies and randomized controlled trials are still lacking to establish a biologic foundation for the use of most of the available materials and agents for reaching true periodontal regeneration [15].

One of the prevalent side effects of periodontitis is spacing between the affected teeth due to tooth movement or pathologic tooth migration (PTM) [16]. This may be esthetically and functionally disturbing, especially in the anterior region. Orthodontic treatment is one of the options to close such spaces, but is only advised after successful treatment of the periodontal condition as part of a collaborative team approach [16].

The following case illustrates how non-surgical periodontal therapy (NSPT) was associated with natural closure of a chronic space present between two periodontally involved anterior teeth, without the use of any external devices. Ethical requirements of the Health Insurance Portability and Accountability Act (HIPAA) of 1996 were followed and all patient-related data were securely hidden in this case, which was reported according to the CARE guidelines for case reports.

## 2. Case Presentation

### 2.1. Chief Complaint, Habits and Health Status

This case concerns a 34-year-old woman, who is currently in good general health, and had quit smoking 3 years ago after 15 years of continuous heavy smoking of 20 cigarettes per day. She visits her dentist regularly and occasionally receives tooth cleaning. She started complaining of a space between her upper front teeth that was getting bigger over the past year, and wanted orthodontic treatment to fix the problem. Her dentist advised her to seek professional periodontal evaluation prior to any orthodontic intervention. Further investigation at the periodontics specialty clinic revealed that she suffered from bleeding gums upon regular tooth brushing and that she had never received specialized periodontal therapy. She also mentioned that her grandfather had lost all his teeth at the age of 40. No tooth-clenching, tongue-thrusting or mouth-breathing habits were reported at this point.

### 2.2. Intra-Oral Clinical Findings

Upon clinical examination, the patient had all her teeth present, generalized gingival inflammation, bulbous and shiny interdental papillae, areas of gingival recession and multiple tooth fillings (Figure 1). No signs of occlusal tooth wear or flaring of teeth were observed. Oral hygiene was generally fair, with a plaque control record (PCR) of 38% according to O’Leary et al. [17]. In addition, a notable space or diastema between teeth # 11 and 12 was observed (Figure 2). Using a periodontal probe, the diastema measured a distance of about 1 mm incisally and 2 mm at the mid-crown level. Initial periodontal pocket (PPD) measurements revealed that 37% of the sites were 4 mm or more in depth, with 19% measuring 6 mm or more, particularly at teeth # 12 and 21 (Figure 3). Bleeding on probing (BoP) reached up to 44%, whereas no tooth mobility or furcation involvements were detected in any of the teeth.

### 2.3. Radiographic Picture

Full intra-oral periapical and bite-wing radiographs showed generalized horizontal bone loss, which was more pronounced in the lower anterior region (Figure 4). In addition, vertical (intrabony) defects were observed at the mesial aspects of teeth # 12, 21, 45 and 46. All initial and follow-up radiographs were taken using alignment devices attached to bite blocks and indicator rings to maintain fixed and reproducible geometric relations.

### 2.4. Diagnosis and Classification

The patient had a right and left molar Class I malocclusion [18]. In addition, the case was periodontally classified as *Localized Periodontitis Stage III Grade C* based on the 2017 World Workshop Classification of Periodontal and Peri-Implant Diseases and Conditions [19]. The advanced stage of the disease was assigned due to the presence of intrabony defects of more than 3 mm in depth that were reaching up to the middle third of the root lengths, and tooth spacing as a result of the disease. The higher grade, on the other hand, was given as the ratio of the percentage of bone loss in the deepest site to the patient’s age, and was more than 1.0. The extent of the disease was assigned since less than 30% of the tooth sites were involved [19]. The intrabony defects were 2- to 3-walls in architecture [20]. All teeth, including those with closely associated defects, were initially projected to be maintainable with a favorable overall prognosis, provided that the formulated treatment plan is followed, and no unanticipated complications would occur.

### 2.5. Treatment Plan and Progress

The set aim of the treatment was to arrest the progression of the active periodontal disease condition and improve periodontal health. Maintenance of the current dentition was also a priority. Any other treatment interventions such as orthodontic tooth movement were not to be discussed till the initial phase of therapy had concluded.

Therapy started by educating the patient regarding the nature of periodontal disease, the complexity in which different patient-, environment- and site-level factors interact and contribute to its development and progression, and the vital roles that both the clinician and the patient have in treating the condition [7,8]. This was followed by discussing the best oral hygiene (OH) practices for the patient, including the choice of a soft personal toothbrush, a suitable toothbrushing method, the use of interdental cleaning aids, i.e., interdental brushes, the frequency of daily toothbrushing and clearance of any misconceptions regarding the negative impact from toothbrushing. The use of mouthwash or other additional chemical agents was discouraged at this phase considering the young age of the patient, her good manual dexterity, and the anticipated favorable outcome from the mechanical treatment. Moreover, using chemical agents early during treatment may mask the effectiveness and compliance of the patient’s OH practices, which should be carefully evaluated at this stage.

Non-surgical periodontal therapy (NSPT) was initiated under appropriate local anesthesia, following a quadrant strategy, i.e., one mouth quadrant per visit, and utilizing a combination of hand instrumentation and ultra-sonic machinery. All accessible supra- and sub-gingival plaque and calculus deposits were meticulously removed, and post-operative instructions were then given. The patient was told to expect shrinkage of the gingiva that occurs naturally following resolution of the inflammation. As a consequence, she might feel that her teeth became longer and that there are so-called black triangles between some of the teeth.

Two months after completing the initial NSPT, the periodontal and overall status of the patient were re-evaluated. No change in medical history or related habits was recorded. Intra-orally, the gingiva appeared healthier, with a natural pale pinkish color (Figure 1), and as expected, gingival shrinkage was observed at some sites, accompanied by gingival recession and black triangles. Surprisingly, tooth # 12 appeared to have moved back into its original position, along with complete closure of the diastema between teeth # 12 and 11 (Figure 2). In addition, significant improvements in the PCR, BoP and PPD had occurred (Figure 3). However, persistent deep pockets of ≥5 mm in depth were observed, particularly at the mesial aspects of teeth # 16 and 21. Such sites were also positive for plaque and BoP. A new radiographic image showed apparent filling of the intrabony defects mesial to teeth # 21 and 12 compared to the initial visit (Figure 5).

At this stage, it was clear that further treatment was necessary in order to control the remaining pathology. However, the decision was to re-treat the diseased sites non-surgically. Surgical intervention was not indicated since the PCR and BoP were still relatively high, and promising findings can be anticipated with reinforcement of OH measures, particularly at those specific sites, especially since the patient showed a high level of compliance with home instructions during the past period.

Accordingly, selective NSPT was carried out at the bleeding sites with deep PPD in a fashion similar to that described for the initial treatment, but due to scheduling conflicts, re-evaluation of the case upon completion could not take place until after the summer vacation, i.e., approximately 4 months after completing the re-treatment phase. Fortunately, the patient was very compliant with the OH home instructions when she was away, and further improvements in clinical parameters such as the PCR, BoP and PPD, were observed on re-evaluation (Figure 3).

### 2.6. Outcome and Maintenance Care

The patient reported being very satisfied with the final treatment outcome functionally and esthetically (Figure 1). She felt that her teeth were now healthy, and she was happy that orthodontic treatment was no longer needed to close the space that was present between her upper front teeth when she first arrived at the clinic. Furthermore, she was not interested in any plastic periodontal surgical options to fill the interdental space between the upper front central teeth. As the active therapeutic phase concluded, the patient was put under supportive periodontal care (SPC) and was recalled on specific interval periods depending on her periodontal status and other contributory factors. The periodontal risk assessment (PRA) model dictated that the patient was of medium risk of progression of the periodontal condition at that stage [21]. Accordingly, she was put on a customized recall program every 6 months (Figure 6).

During the first recall visit scheduled 8 months later, the periodontal condition appeared stable following the revision of all background, health and clinical parameters (Figure 1 and Figure 3). A new radiograph for tooth # 21 also suggested further apparent filling of the intrabony defect mesial to tooth # 21 (Figure 5).

In total, the active treatment lasted for 7 months. Following the initial visit, NSPT was carried out (1 month), followed by the first re-evaluation (2 months). The second re-evaluation was then done after re-treatment by non-surgical means (4 months). Afterwards, the patient was put under a dynamic personalized SPC protocol, with recalls every six months depending on her risk level.

## 3. Discussion

The presented case is for a 34-year-old woman, who requested orthodontic closure of a diastema between her upper front teeth, but was advised by her general dentist to seek specialized periodontal evaluation first. Since the tooth movement causing the space was associated with advanced periodontal disease, and given the apparent absence of other contributory factors, comprehensive periodontal treatment planning and therapy ultimately addressed the patient’s complaints. She was also satisfied with complete closure of her diastema. This aligns well with the earlier literature demonstrating how reactive tooth positioning, i.e., teeth returning to their normal positions without any external devices or forces, may spontaneously occur following successful treatment of the periodontal condition [22]. Gaumet et al. [23] and Thakur and Baburaj [24] analyzed a number of moderate to severe periodontitis patients who suffered from diastemas secondary to pathologic tooth migration, and reported that a higher percentage of diastemas closed completely after surgical periodontal therapy, particularly those that were small to moderate in size (<1 mm). Interestingly, the diastema in the current case was >1 mm incisally and 2 mm mid-crown level, and was treated non-surgically. This case also illustrated the importance of establishing a productive and accessible referral system between the general practitioner and the periodontal specialist, and overcoming the obstacles that lead to low-quality or no referrals [25,26], which in turn may delay a strategic decision that is crucial for a favorable patient outcome. Based on a recent review, and as the current case suggests, treating the active periodontal condition prior to considering any orthodontic intervention proved to be the correct decision, and should generally be assessed on a case-by-case basis [27].

The current case also demonstrated how effective non-surgical periodontal therapy (NSPT) resulted in improvement of the periodontal condition, and was associated with apparent filling of the intrabony defect as suggested in the radiographic images, and is aligned with what is reported in the classic literature [28]. In fact, Nibali et al. [29] observed a significant reduction in intrabony defects radiographically 12 to 18 months post-treatment. Generally, response to NSPT is more pronounced in individuals with initially severely active periodontal disease, and less favorable in current heavy smokers as suggested by Raittio and co-workers [30].

It has been suggested that non-surgical re-treatment of residual periodontally involved sites (PPD ≥ 5 mm) adds the benefit of better visualization and accessibility to missed/remaining calculus deposits following shrinkage of previously inflamed gingival tissues [31]. This may also give patients a chance to improve their self-performed oral hygiene practices. However, Badersten et al. [32] had classically stated that repeated non-surgical treatment of residual periodontal pockets did not yield any further clinical improvements than those achieved following the initial NSPT. Nevertheless, multilevel analyses revealed that careful assessment of various individual-, site- and operator- related factors should precede the decision to go for non-surgical re-treatment. An individual’s attitude towards compliance with oral hygiene measures is key for a successful outcome, while periodontal pockets associated with molars, furcations and intrabony defects respond less favorably to re-instrumentation attempts [33]. In the current case, the decision to re-treat the remaining periodontally diseased sites non-surgically proved to be a correct one as further clinical and radiographic improvements of those sites, including those associated with the intrabony defects, were observed over time. Furthermore, going for surgical intervention in situations where the plaque and bleeding indices are still at the upper limits is considered a contraindication and should be deferred till such parameters are lowered to an acceptable level to avoid failure of the procedure [34].

Following successful completion of the active periodontal therapy phase, the patient was put under supportive periodontal care (SPC). This is important to ensure stability of the periodontal condition over time. Studies have demonstrated the crucial role that SPC plays in reducing the incidence of tooth loss [35]. Individuals who do not comply with SPC are at a higher risk of recurrence of the disease and losing teeth than those who do comply [36]. Smoking, diabetes, age, frequency of daily toothbrushing, severity of the periodontal condition, complexity of the provided treatment, and dental caries are among the factors associated with incidence of periodontal disease progression and tooth loss [37,38]. Another review went even further and stated that long-term maintenance of periodontally treated teeth is more cost-effective than replacing them with dental implants [39]. Additionally, the periodontal risk assessment (PRA) model was utilized to develop a customized maintenance program for the current patient rather than providing one with a fixed set of procedures and a standard interval between visits. This was an important step to increase the effectiveness of such a program. A multi-center randomized controlled trial concluded that no significant differences could be observed between fixed recall intervals and risk-based intervals in terms of clinical oral health parameters [40]. However, risk-based intervals were more cost-effective and appreciated by the patients. Generally, short-interval visits have been associated with a lower risk for disease recurrence than visits with longer intervals between them [41].

## 4. Limitations

The current report documents a single case of an anterior diastema that may have resulted from pathologic tooth migration and was resolved following non-surgical periodontal therapy. In addition, lack of histological data and a comparative (control) case clearly limit the generalizability of the findings. Nevertheless, and although such a concept has been reported in the literature, the combination of high patient compliance in oral hygiene habits and quitting smoking, good general dentist-to-specialist coordination and thorough treatment planning and execution gave an added value to this report, with potential areas for future research. Another limitation is the lack of quantitative clinical and radiographic measurements for the diastema and defects changing over time. Similarly, the absence of longitudinal CAL records around the affected teeth also necessitates the interpretation of the results with caution. It is noteworthy that site-specific CAL measurements were not recorded at the time as no decision for reconstructive surgical procedures was taken.

## 5. Conclusions

Within the limits of this report, this single case illustrates the short-term effectiveness of non-surgical periodontal therapy, in the presence of good patient compliance and continued maintenance care, in the resolution of the periodontal condition. The case also cautiously highlights the possible association with another favorable outcome in the form of movement of the affected teeth back into their normal positions and accordingly, addressing the patient’s esthetic concerns without the need for orthodontic treatment. However, the predictability of achieving such an outcome needs to be confirmed in studies with a larger-scale and standardized settings, with detailed documentation of longitudinal data.

Key takeaways include that non-surgical periodontal therapy is the basic, evidence-based, cause-related treatment modality that should first be executed effectively when treating periodontal disease. Surgical treatment modalities are viable options that are equally effective, but should only be considered when indicated, when the non-surgical phase is complete, and persistent pathology is still there. Customized maintenance care is as important as the active therapeutic phase and should not be overlooked in order to ensure periodontal stability and health. Positive collaboration between the general practitioner and the specialist is of utmost importance to address the patient’s needs in a timely and efficient manner. Officials and concerned policymakers should establish a smooth and effective referral system to make use of the efforts and expertise of the healthcare workforce and available resources.

## Figures and Tables

**Figure 1:**
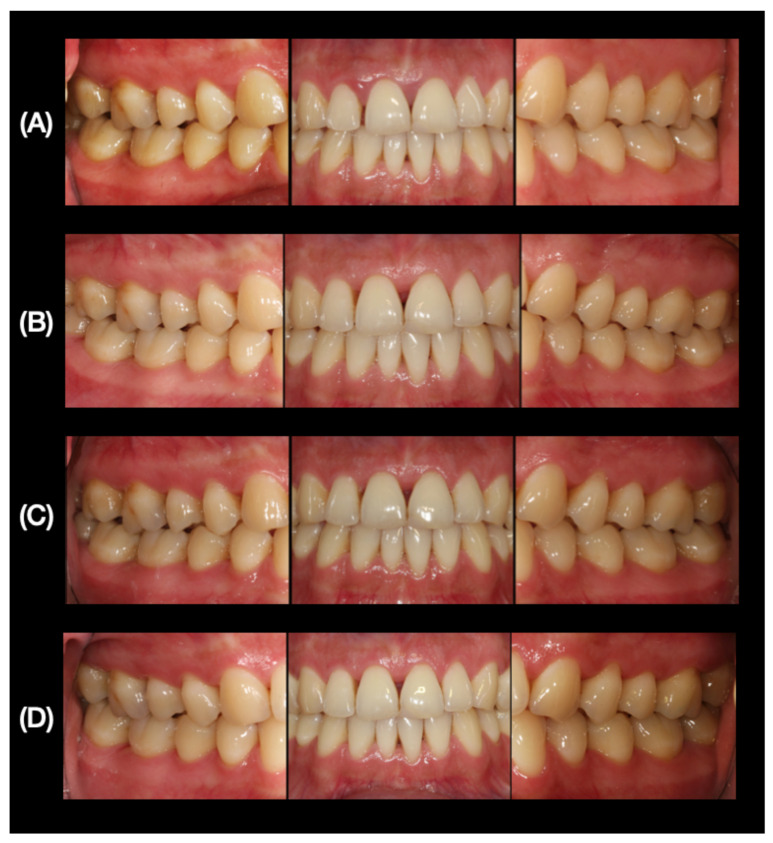
Right, frontal and left clinical photographs at the (**A**) initial, (**B**) 1st re-evaluation (2 months post-treatment), (**C**) 2nd re-evaluation (4 months post-treatment), and (**D**) 1st recall visits (8 months post-treatment).

**Figure 2:**
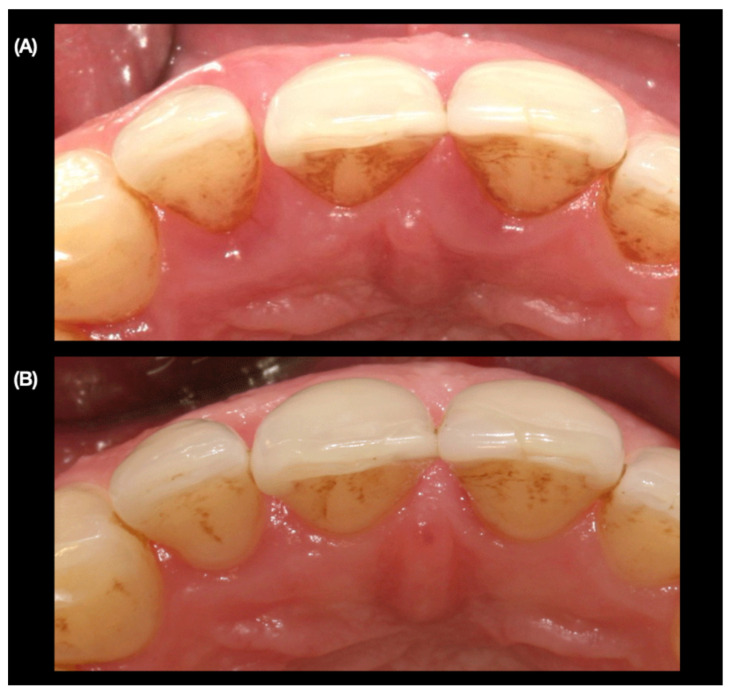
Occlusal views of the diastema between teeth # 12 and 11 at the (**A**) initial, and (**B**) 1st re-evaluation visits (2 months post-treatment).

**Figure 3:**
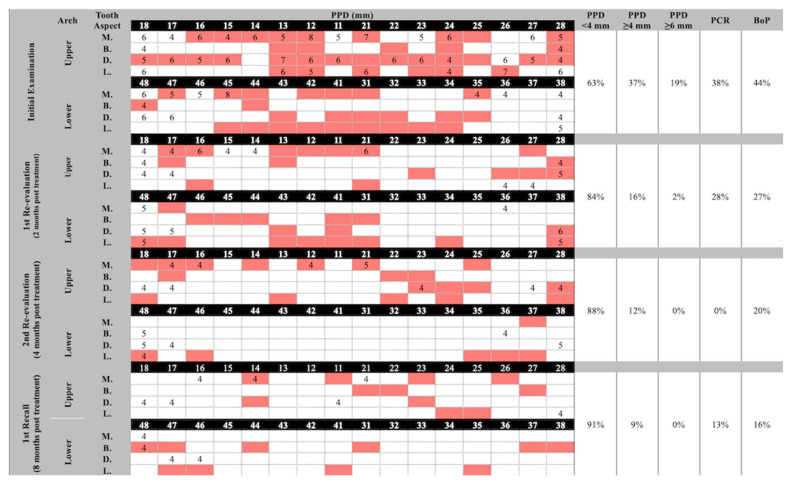
Periodontal measurements at the initial, re-evaluation and recall examinations. A clear decline in probing depth, plaque and bleeding scores was observed over time, representing improvement in periodontal status and patient compliance. (PPD: Probing Pocket Depth, M.: Mesial, B.: Buccal, D.: Distal, L.: Lingual, PCR: Plaque Control Record, BoP: Bleeding on Probing. Highlighted cells represent periodontal pockets with bleeding on probing. Empty cells are pockets with PPD < 4 mm).

**Figure 4:**
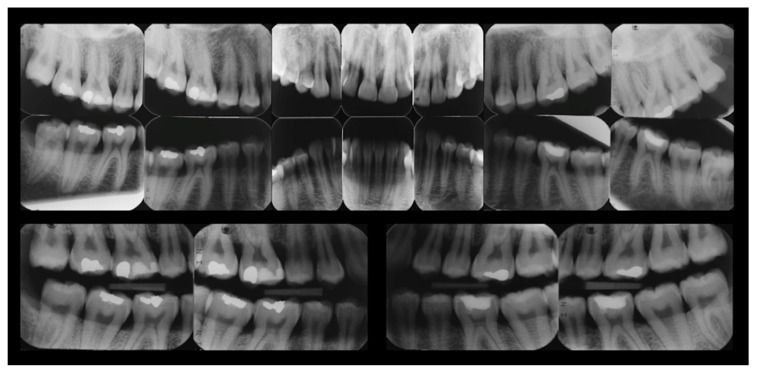
Pre-treatment full mouth intra-oral radiographs.

**Figure 5:**
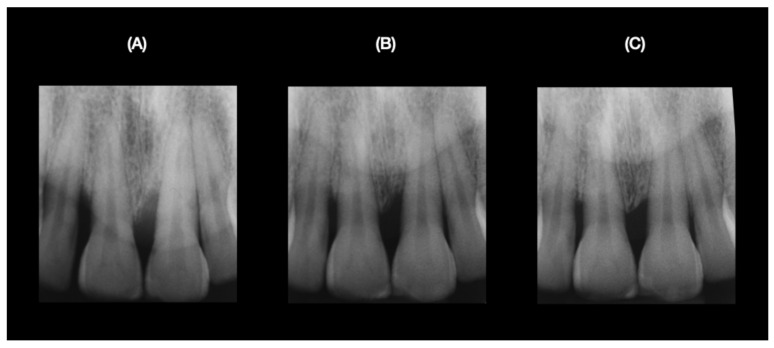
Periapical radiographs of intra-bony defects at teeth # 12 and 21 at the (**A**) initial, (**B**) 2nd re-evaluation, and (**C**) 1st recall visits.

**Figure 6:**
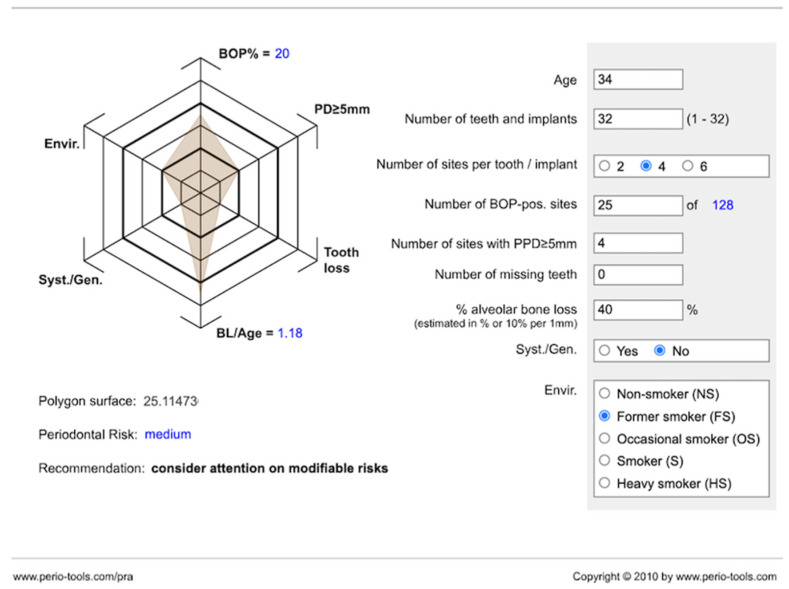
Periodontal risk assessment (PRA) model showing medium periodontal disease risk of progression following completion of the active phase of periodontal therapy and prior to designing a suitable maintenance program for the patient.

## Data Availability

Data is unavailable due to privacy restrictions.
